# The Insurance of Motherhood

**Published:** 1912-03-30

**Authors:** 


*To the Editor of* The Hospital.

### THE INSURANCE OF MOTHERHOOD.

An Explanation of the Maternity Benefit of the Insurance Act.

*To the Editor of* The Hospital.

A Solicitor writes: In comparison with men, few women make provision for sickness; and neither men nor women have much knowledge of insurance for the expenses of child-birth. By reason of its novelty, for its importance and interest, and because of the difficulty women may find in disentangling the meaning of the crabbed legal phrases of the Act, information in as simple a form as possible on this benefit alone may well be given. The usual contribution for all the benefits of the Act for women will be 6d. per week, payable by the employer, who may deduct 3d. of it from the wages. The usual benefits will be medical treatment and medicines; sanatorium treatment for consumption; a payment of 30s. on confinement; and a sickness benefit of 7s. 6d. weekly for twenty-six weeks and 5s. afterwards until the age of seventy is reached. This latter benefit will be always lower for those under twenty-one years of age, and will be lower for those over fifty years of age for some years after the Act is in operation.

During the first year after the commencement of the Act working women of all ages will be taken into insurance at one contribution of 6d. per week, but after this period the normal case will be that such women will enter into insurance at sixteen years of age, for there is no compulsory insurance below that age. They will pay for the few years of youthful employment and then marry. On marriage a woman's payments cease, and she becomes suspended from all the ordinary benefits until the death of her husband, unless she continue her employment, in which case the contributions continue and benefits will be the same as before marriage. If she ceases to be employed she has still some small benefits to receive. Though payments by the employer and herself will have ceased, she will receive on confinement 5s. weekly for four weeks. If, however, she wishes to continue her insurance voluntarily, she may continue to pay her 3d. per week, and will receive medical treatment and a sickness benefit of 5s. for the first thirteen weeks of sickness and 3s. afterwards. The sick benefit will not be paid during the two weeks before and the four weeks after confinement.

It may be pointed out that an insured man, as he pays the contributions, receives the maternity benefit himself from his society when his wife is confined, and whether she is insured or not; a woman also only gets this benefit herself when she is insured. She may receive maternity benefit from her society as a single woman, a wife, or a widow. In no case, though both husband and wife work and are insured, can two maternity benefits be claimed, but it will be seen later that provision is made for substituting a sickness benefit where there would otherwise be two maternity benefits, both husband and wife being insured.

1. The most common case is that of a working-man whose wife has been employed before marriage but has ceased employment on marriage. On her confinement the husband will receive 30s. from his society in cash or otherwise as the society decides. It is to be hoped that societies will give it "otherwise," and prevent certain husbands from using the money to celebrate the event. No benefit can be mortgaged, nor can it be used as a security for borrowed money. If the wife ceased to pay contributions on marriage, she will receive in addition and from her society the special sick benefit of 5s. for four weeks. If she continued to pay 3d. per week she will get nothing in addition to her husband's benefit, the 3d. a week she has paid having been devoted to other benefits than maternity.

2. Next we have the case where both husband and wife continue to be employed after marriage, as is common in Lancashire. Here, again, both are insured, and the man will receive 30s. from his society, while the woman would get 7s. 6d., the ordinary weekly sickness benefit from her society, for so long as her sickness lasts, in substitution for her maternity benefit.

3. Another very common case may be dealt with where the wife has been at home before her marriage or was not insured under the Act for some other reason when she married. On her confinement the husband, if insured, would receive 30s., or its value; the wife would get nothing.

4. Where, however, it is the wife alone who is insured, and the husband not, strange as it may seem, though only one contribution has been paid, the wife will get two benefits from her society—the 30s. maternity benefit and the 7s. 6d. per week sick benefit, so that the additional sickness benefit will permit her to remain away from work some weeks at little pecuniary loss.

5. An unmarried insured woman will receive 30s. only and no sick benefit until after four weeks from her confinement.

6. And a widow who has a child to her husband after his death will get just such benefits as she would have had had her husband lived.

One may wonder what will become of the contributions while an employed woman is away from work on confinement and unable to pay; would her employer be paying sixpences for her and piling up a debt against her return to work? He would not. No payments need be made by the employer or the woman, nor need she pay anything on her return to work, for her employer will be excused payment of contributions while she is unpaid and away from work. On her part no arrears of contributions will accrue against her during two weeks before and four weeks after the confinement, and it may be added that a widow will be excused all arrears of contributions during the period after the father's death and until the birth of the child.

It has been necessary to provide punishment for a husband who neglects to make adequate provision for the care of his wife during her confinement, though he has paid to him money for the purpose. Provisions are to be found in the Insurance Act whereby a month's imprisonment with hard labour may be awarded to such a callous husband. The fear of punishment will no doubt have the effect of preventing wasteful expenditure of the maternity money, if his society allows the husband to handle it.

In every case the mother is given free choice in selecting her doctor or certificated midwife. The mother may have either or both and whom she pleases, and if such a nurse be chosen who finds that a doctor is necessary, a doctor may be called in and his fee paid as part of the maternity benefit.

As the money payment on maternity is intended to provide medical attendance, the ordinary medical benefit will be suspended when maternity benefit is received. A word might be added with regard to those frequent cases where a woman goes into a hospital or infirmary to have her child. She cannot be refused admission to a workhouse infirmary solely because she has maternity benefit. Where she goes into a workhouse, public hospital, or one supported by charitable subscriptions, she will again cease to receive maternity benefit, but her society must use it for the maintenance of anyone she may have dependent in part on her earnings. Should she have no dependants, then the benefit may be paid to any such hospital or convalescent home she may be in towards her maintenance there. Apart from hospital treatment, societies may provide district nurses and convalescent homes for their members out of the State funds, and it need hardly be added that the societies' sick visitors must be women.

Little need now be said to point out the far-reaching effects of the maternity benefit. The reader will have noticed how carefully provision has been made in all cases to enable a woman to be away from work for a proper period before and after confinement. There are no payments of contributions to make, no arrears incurred, and money for the necessary expenses is found. Strong hopes are entertained that infant mortality will decrease, and that the evil effects of women's work near this critical period will gradually cease.

